# New insights into signal transduction pathways in adrenal steroidogenesis: role of mitochondrial fusion, lipid mediators, and MAPK phosphatases

**DOI:** 10.3389/fendo.2023.1175677

**Published:** 2023-05-08

**Authors:** María Mercedes Mori Sequeiros Garcia, Cristina Paz, Ana Fernanda Castillo, Yanina Benzo, Matías A. Belluno, Ariana Balcázar Martínez, Paula Mariana Maloberti, Fabiana Cornejo Maciel, Cecilia Poderoso

**Affiliations:** ^1^ Universidad de Buenos Aires, Facultad de Medicina, Departamento de Bioquímica Humana, Buenos Aires, Argentina; ^2^ CONICET-Universidad de Buenos Aires, Instituto de Investigaciones Biomédicas (INBIOMED), Buenos Aires, Argentina

**Keywords:** adrenocortical cells, mitofusin 2, oxoeicosanoids, MKP-3, protein kinases, steroidogenesis, mitochondrial fusion, OXER1

## Abstract

Hormone-receptor signal transduction has been extensively studied in adrenal gland. *Zona glomerulosa* and *fasciculata* cells are responsible for glucocorticoid and mineralocorticoid synthesis by adrenocorticotropin (ACTH) and angiotensin II (Ang II) stimulation, respectively. Since the rate-limiting step in steroidogenesis occurs in the mitochondria, these organelles are key players in the process. The maintenance of functional mitochondria depends on mitochondrial dynamics, which involves at least two opposite events, i.e., mitochondrial fusion and fission. This review presents state-of-the-art data on the role of mitochondrial fusion proteins, such as mitofusin 2 (Mfn2) and optic atrophy 1 (OPA1), in Ang II-stimulated steroidogenesis in adrenocortical cells. Both proteins are upregulated by Ang II, and Mfn2 is strictly necessary for adrenal steroid synthesis. The signaling cascades of steroidogenic hormones involve an increase in several lipidic metabolites such as arachidonic acid (AA). In turn, AA metabolization renders several eicosanoids released to the extracellular medium able to bind membrane receptors. This report discusses OXER1, an oxoeicosanoid receptor which has recently arisen as a novel participant in adrenocortical hormone-stimulated steroidogenesis through its activation by AA-derived 5-oxo-ETE. This work also intends to broaden knowledge of phospho/dephosphorylation relevance in adrenocortical cells, particularly MAP kinase phosphatases (MKPs) role in steroidogenesis. At least three MKPs participate in steroid production and processes such as the cellular cycle, either directly or by means of MAP kinase regulation. To sum up, this review discusses the emerging role of mitochondrial fusion proteins, OXER1 and MKPs in the regulation of steroid synthesis in adrenal cortex cells.

## 1 Introduction

Adrenal steroid hormones modulate a wide range of processes which are central to the physiological response to stress, including energy metabolism, immunity, electrolyte homeostasis, and fluid balance. In humans, *zona glomerulosa* (ZG), *zona fasciculata* (ZF), and *zona reticularis* (ZR) are the histologically distinct adrenal cortex regions responsible for the production of mineralocorticoids, glucocorticoids, and androgens, respectively. The main signaling pathways in hormone regulation in the adrenal cortex, extensively described as the “classical” pathways, are evoked by adrenocorticotropin (ACTH), K^+^, and angiotensin II (Ang II) (1).

Adrenal steroid synthesis is mainly controlled by ACTH in ZF and by Ang II, K^+^ and ACTH in ZG. ACTH activates the melanocortin 2 receptor (MC2R) (2), a G-protein-coupled receptor (GPCR) which promotes cAMP production and activates protein kinase A (PKA) (3). Ang II binds to the membrane angiotensin receptor type 1 (ATR1) (4, 5), a GPCR that increases diacylglycerol (DAG) and inositol trisphosphate (IP_3_) production, which in turn promotes an increase in cytosolic Ca^2+^. This leads to the activation of several isoforms of protein kinase C (PKC) (3), a family of serine/threonine kinases widely implicated in the control of cell growth, motility, and survival. In turn, PKCs activate a wide spectrum of targets, including other kinases such as protein kinase D or PKCµ, involved in steroidogenesis (6). In addition, steroidogenic hormone cascades include the activity of mitogen-activated protein kinases (MAPKs), a group of serine/threonine kinases comprising ERKs, JNKs, and p38. Both cAMP/PKA- and IP_3_/DAG-Ca^2+^/PKC-dependent pathways ultimately enable the activation of the rate-limiting step in steroid production, i.e., cholesterol transport from the outer (OMM) to the inner mitochondrial membrane (IMM), where it is converted to pregnenolone. This crucial step is mediated by steroidogenic acute regulatory protein (StAR),a key mitochondrial protein (7, 8). One of the most widely used models of adrenal cortex functions is the human adrenocortical carcinoma cell line H295R. This cell line is a substrain derived from NCI-H295 cells, which were originally isolated from the adrenal gland of a 48-year-old female adrenal cancer patient (9). H295R cells respond to Ang II, ACTH/cAMP and K^+^ by increasing the production of aldosterone and other steroids (10).

In harboring the first step in the synthesis of all steroid hormones, mitochondria are essential in highly specialized steroidogenic cells. Mitochondrial functions have been associated with their capacity to undergo fusion and fission processes which lead to modifications in morphology and movement throughout the cytoskeleton. High molecular weight GTPases, mitofusin (Mfn) 1 and 2, and optic atrophy 1 (OPA1) are essential modulators of mitochondrial fusion in mammals, whereas dynamin-like related protein 1 (Drp1) participates in mitochondrial fission. Although ultrastructural morphological changes have long been observed in the mitochondria of hormone-stimulated adrenal gland cells (11), a possible role of mitochondrial fusion/fission in steroidogenesis has more recently emerged in the study of adrenal cortex function.

Lipid metabolism is a complex pathway involving several heterogeneous compounds, as lipids display a wide range of chemical structures. One of the lipidic pathways involved in adrenal cortex steroidogenesis, arachidonic acid (AA) synthesis is principally mediated by acyl-CoA synthetase type 4 (ACSL4), which has high specificity for this long chain fatty acid (12). AA is known to take part in StAR gene induction and, thus, steroidogenesis in murine MA-10 Leydig and H295R cells (13). Among other AA-derived metabolites participating in hormonal signaling, oxoeicosanoids activate their membrane receptor OXER1 in autocrine and paracrine manners and participate in steroidogenesis in H295R cells (14, 15).

Adrenal cortex signal transduction also involves protein phospho/dephosphorylation cascades. Many relevant tyrosine and serine/threonine phosphatases such as SHP2 and PP1 participate in hormone-stimulated steroidogenesis (13, 16–21). Another group includes dual protein phosphatases which exhibit specificity for MAPKs and are hence called MAPK phosphatases (MKPs) (22). MKPs are involved in steroidogenesis in both human adrenocortical H295R and murine Leydig cells through ERK1/2 dephosphorylation and regulation (23–25). Recently, Ang II-upregulated MKP-3 has been associated with the cellular cycle in H295R cells (25).

In this context, this review discusses the role of emerging molecular pathways in the regulation of essential biological processes such as steroid biosynthesis.

## 2 Hormone-regulated mitochondrial fusion plays a role in adrenocortical steroid synthesis

Research on mitochondria reorganization and morphology in steroidogenic tissues dates back to the 1970s. Bornstein and colleagues showed an increase in mitochondrial volume and dense vesicularization of mitochondrial *cristae* in the rat adrenal gland after corticotropin-releasing hormone administration, which may constitute an early step in enhancing cellular steroidogenic capacity (11). Interestingly, over a decade ago Li and Sewer proved a novel mechanism through which ACTH regulates mitochondrial movement in H295R cells (26), one of the first discoveries that mitochondria are not merely static organelles in steroid biosynthesis. Mitochondrial fusion and fission regulate mitochondrial network connectivity and have high impact on the specific metabolic requirements of each cellular type (27, 28). Fusion proteins Mfn1 and Mfn2 are located on the OMM, although Mfn2 is also present on the endoplasmic reticulum membrane. In addition, IMM fusion requires OPA1 exposure to the intermembrane space. Regarding fission proteins, Drp1 is mainly located throughout the cytosol, and a fraction of Drp1 localizes to the mitochondrial focal points indicating future fission sites (29). Worth pointing out, the impairment of mitochondrial function has been associated with the development of several diseases (30–32).

In agreement with previous work in murine MA-10 Leydig cells (33, 34), our group has recently explored the notion that mitochondrial fusion may ensure full steroidogenesis in adrenocortical cells. We showed for the first time that Ang II upregulates mitochondrial fusion, as observed through immunofluorescence in H295R cells. Ang II promotes a switch from punctuated toward more elongated mitochondrial morphology −indicative of fusion−, and Mfn2 expression increases after Ang II and K^+^ treatment in a time-dependent manner (35). Nevertheless, whether Mfn2 is regulated at a transcriptional and/or post-transcriptional level remains to be established and further studies on transcription factors regulating Mfn2 may prove of interest. Remarkably, Mfn2 has a key role in H295R cell steroidogenesis, as its silencing by knock-down exerts an inhibitory effect on Ang II-stimulated aldosterone synthesis (35).

Another mitochondrial fusion protein, OPA1 participates in mitochondrial Ca^2+^ handling, particularly restraining Ca^2+^ uptake (36), and is regulated by Ang II in H295R cells (35). OPA1 modulation involves alternative splicing, which renders at least eight mRNA variants in humans and generates long (L-OPA1) and short (S-OPA1) isoforms (37). Ang II favors early L-OPA1 isoforms, whereas S-OPA1 is observed at later time-points in H295R cells. In addition, L-OPA1 seems to be required for IMM fusion processes, while some S-OPA1 variants are dispensable (38). Therefore, a balance between long and short isoforms over Ang II-stimulation time may promote mitochondrial fusion (35). As regards fission, Ang II favors a decrease in mitochondrial Drp1 levels, a widely described anti-fission event, with a concomitant balance toward mitochondrial fusion (35).

In line with our previous results (33), we have demonstrated in H295R cells that Mfn2 expression is strictly required for the mitochondrial activation of ERK1/2 and its upstream kinase MEK1/2 after Ang II stimulation. Ang II effects on mitochondrial MEK and ERK rely on kinase phosphorylation rather than on total kinase levels. Interestingly, Mfn2 expression is necessary for StAR mitochondrial association even when the StAR sequence bears a mitochondrial signaling peptide. As previously mentioned, Mfn2 knock-down decreases steroidogenesis in H295R cells (35), which may be at least partly attributed to the role of Mfn2 in MEK, ERK and StAR mitochondrial localization. Although the molecular mechanisms underlying this process remain to be tested in adrenocortical cells, mitochondrial fusion through Mfn2 expression and OPA1 and Drp1 regulation is strictly required for Ang II steroid synthesis stimulation in H295R cells.

## 3 Lipid mediators and the oxoeicosanoid receptor 1 (OXER1) regulate adrenocortical cells steroid production and migration

Hormonal stimulation of steroid synthesis in ZF and ZG cells, as well as in testicular Leydig cells, entails AA release (39). AA is metabolized via the lipoxygenase (LOX) (40, 41), epoxygenase (42), and cyclooxygenase (43) pathways to produce a range of bioactive metabolites. LOX products of AA metabolism can be detected in rat and bovine cultured adrenal and rat Leydig cells following stimulation with ACTH, Ang II and luteinizing hormone (LH) respectively (44–47). The LOX pathway transforms AA into hydroperoxyeicosatetraenoic acids, hydroxyeicosatetraenoic acids and oxoeicosatetraenoic acids (HpETEs, HETEs and oxo-ETEs, respectively) (44). 5-HETE and 5-HpETE, produced by the action of 5-lipoxygenase (5-LOX), exert a positive effect on steroid biosynthesis by stimulating StAR promoter activity (48). 5-oxo-ETE is produced through 5S-HETE oxidation by selective enzyme 5-hydroxyeicosanoid dehydrogenase (5-HEDH) (49). Regarding these metabolites, OXER1 is an oxoeicosanoid receptor of the leukotriene receptor family belonging to the GPCR superfamily (50–52) whose cloning, putative agonists, cellular expression (50, 51, 53–56) and function (52, 55–58) have only been scarcely documented over the last two decades. 5-HpETE and 5-oxo-ETE are OXER1 ligands with the highest affinity and triggering the strongest response. OXER1 expression has been detected in human steroidogenic tissues (adrenal, testis, ovary, placenta) (50), eosinophils, neutrophils, monocytes, basophils, and macrophages (52, 56, 57), and in cultured prostate, breast, ovarian, and kidney cancer cells (54, 55, 58). OXER1 expression also correlates with the functional effects reported for oxo-derivatives of AA, especially 5-oxo-ETE, in these cell types. These studies suggest that OXER1 participates in inflammatory and allergic responses and contributes to the growth and spread of human tumors.

Given the regulation of StAR protein expression by the LOX products of AA (48), our group studied OXER1 participation in physiological processes such as steroidogenesis, cell proliferation and migration. First, the results revealed the presence of OXER1 in H295R cells. Through a pharmacological approach and the application of molecular biology tools (14, 15), our work also demonstrated that the activation of cAMP-dependent and independent pathways promotes StAR protein induction and steroidogenesis through a process that involves, at least in part, the autocrine or paracrine activation of OXER1, which implies an increase in ERK1/2 phosphorylation. Increasing evidence hints at the involvement of 5-oxo-ETE and OXER1 in cell proliferation and migration (59, 60). 5-oxo-ETE promotes cell growth and survival in breast and prostate cancer cells (55, 58), while OXER1 silencing reduces cell viability (54). In our studies, although 5-oxo-ETE did not affect H295R cell growth, OXER1 overexpression caused an increase in cell proliferation which was further boosted by 5-oxo-ETE and blocked by 5-LOX inhibition. 5-oxo-ETE also increased the migratory capacity of H295R cells but failed to induce the production of metalloproteases 1, 2, 9 and 10. 5-oxo-ETE caused significant activation of ERK and p38, while its pro-migratory effect was reduced by pharmacological inhibition of MEK/ERK1/2, p38 and PKC. ERK activation by this oxoeicosanoid was reduced by pan-PKC inhibitor GF109203X but not by classical PKC inhibitor Gö6976, which suggests the involvement of novel PKCs in this effect. Even though H295R cells exhibit phosphorylation of serine 299 in PKCδ, a readout for this novel PKC activation, treatment with 5-oxo-ETE failed to single-handedly induce additional PKCδ activation (61). It may be thus speculated that lipoxygenase products −through autocrine and/or paracrine actions and their binding to OXER1−may be involved in the fine-tuning of cellular functions, supporting general regulation by major players.

## 4 MKPs expression and function in adrenocortical steroidogenic cells

MKPs are dual activity phosphatases which dephosphorylate both phospho-threonine and phospho-tyrosine residues of MAPK family members and thus regulate their activity (62). MAPKs are known to play a key role in cell proliferation, apoptosis, and steroid synthesis, among others, and MKPs may be thus regarded as potential regulators of these processes (63, 64). At least three isoforms of the MKP family have been detected, i.e., MKP-1 (DUSP1), MKP-2 (DUSP4) and MKP-3 (DUSP6), and their expression, regulation and function have been analyzed in steroid-producing cells.

MKP-1 is a nuclear enzyme able to dephosphorylate ERK1/2, JNK1/2, and p38. MKP-1 was the first MKP to be characterized in steroid-producing cells such as adrenocortical cells. Sewer and Waterman demonstrated that a permeable cAMP analog promoted an increase in both MKP-1 mRNA and protein levels in H295R cells (65). Our group showed that ACTH/cAMP also increase MKP-1 mRNA and protein levels in a rapid and transient fashion in murine adrenocortical Y1 cells (66). In addition, increased intracellular Ca^2+^ levels and PKA activation exert synergic effects on ACTH-mediated MKP-1 gene expression, which suggests PKC participation (66). As ACTH triggers JNK and ERK activation (67, 68), it has been postulated that ACTH-dependent MKP-1 induction participates in ERK dephosphorylation (66). Moreover, Sewer and Waterman demonstrated that, through ERK dephosphorylation, MKP-1 regulates the expression of the hCYP17 gene, which encodes 17 α-hydroxylase, in H295R cells. This findings unveil the role of MKP-1 in steroidogenic gene regulation in the adrenal cortex (69). Furthermore, in bovine adrenal glomerulosa cells, Ang II promotes transient ERK and p38 phosphorylation and an increase in MKP-1, mainly due to mRNA stabilization (70). In these cells, MKP-1 overexpression reduces the effect of Ang II on ERK1/2 phosphorylation and aldosterone production (70).

MKP-2 is also a nuclear enzyme displaying broad substrate specificity. It is induced by several stimuli and exhibits slower induction kinetics than MKP-1 in several tissues (63, 71). In an alternative steroidogenic system, LH/cAMP significantly increase mRNA levels in murine MA-10 Leydig cells, leading to MKP-2 protein accumulation through PKA-dependent but ERK-independent post-translational modifications which increase protein half-life (24). Furthermore, MKP-2 heightens the stimulatory effect of cAMP on the induction of CYP11A1, which encodes the P450scc enzyme (24), playing a role in cAMP-dependent steroid production.

MKP-3 is a cytoplasmic enzyme well recognized as a highly specific phosphatase for phospho-ERK1/2 (72). The human DUSP6 gene produces 2 variants of MKP-3: the widely studied full-length or long transcript (MKP-3L) consisting of 3 exons, and the short transcript (MKP-3S), which lacks the second exon. Although the L/S ratio is known to greatly vary across tissues and cells (73), the expression levels and biochemical and functional characteristics of both isoforms remain to be elucidated, particularly in steroidogenic tissues. Given that Ang II triggers ERK1/2 phosphorylation in a transient fashion in H295R cells (74), our group conducted studies on MKP-3L and MKP-3S expression in this cellular model. Our results revealed both variants’ expression in basal conditions and their upregulation by Ang II. Both messengers were rapidly induced by Ang II stimulation, although the expression of MKP-3L mRNA was higher than that of MKP-3S. Ang II also promoted a rapid increase in both proteins’ levels in a time frame compatible with ERK1/2 dephosphorylation (25).

Even when MKP-3 is highly specific for phosphorylated ERK1/2, it has also been reported to interact with and dephosphorylate Forkhead box protein O1 (FOXO1) transcription factor in mouse liver cells (75). This event leads to FOXO1 nuclear translocation and its subsequent activation for the expression of several FOXO1-dependent genes (76–78). In agreement, in adrenocortical H295R cells, Ang II induces transient FOXO1 phosphorylation and translocation to the nucleus and p21 induction, whereas MKP-3 knock-down reduces both FOXO1 translocation and p21 induction (25). Collectively, the preceding data on MKP-3 may foster more thorough studies of MKP-3 regulation of FOXO1 and its putative impact on adrenocortical cell proliferation.

## 5 Discussion and concluding remarks

**Figure 1:**
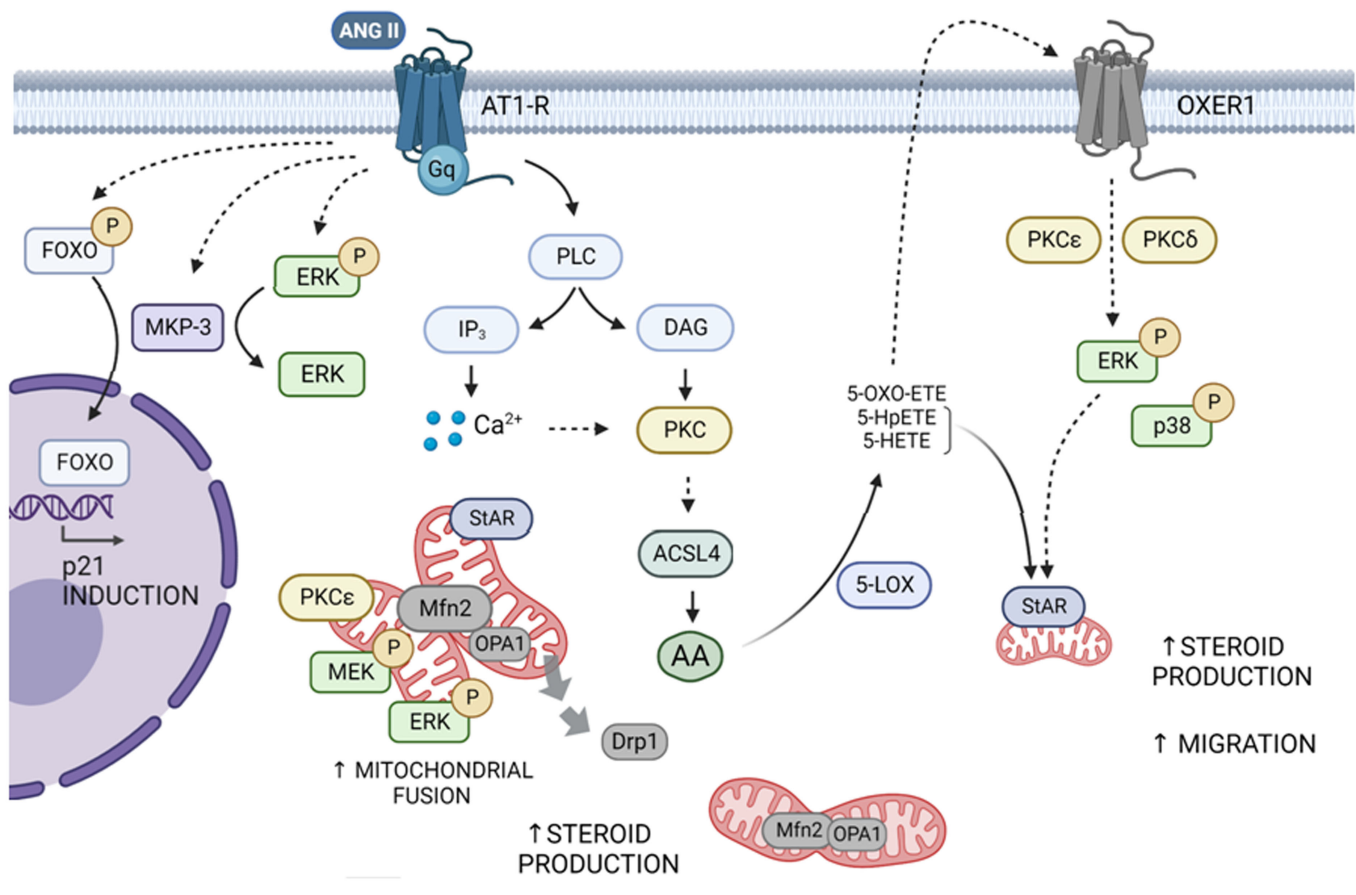
Scheme of the signal transduction pathways triggered by Ang II and OXER1 in adrenocortical cells. Ang II interacts with the AT1R and promotes the activity of PLC which leads to the activation of members of the PKC superfamily, particularly PKCα, ε and δ. Ang II favors the increase in Mfn2 expression, driving mitochondrial fusion. This event enables mitochondrial activation of MEK and ERK and proper localization of StAR, to achieve full steroid production. In line, Drp1 decreases in mitochondria, leaning the balance toward mitochondrial fusion. Through an ACSL4/AA- dependent mechanism, Ang II promotes an increase in oxoeicosanoids. These compounds may activate, in an autocrine and paracrine way, OXER1 on the cell membrane. Signal transduction involves ERK1/2 and p38 phosphorylation dependent on PKCε and δ, and a further increase in StAR expression, steroid synthesis, and cell migration. Simultaneously, activated AT1R by Ang II leads to ERK and FOXO1 phosphorylation and an increase in MKP-3 levels. Later, MKP-3 dephosphorylates p-ERK and p-FOXO1 to promote FOXO1 nuclear translocation and its subsequent activation of p21 protein expression. Dashed and solid arrows indicate indirect and direct activation, respectively.

Although adrenocortical steroid production has been extensively studied and revised, this review discusses some of the latest and less widely described mechanisms involved in hormone regulation of adrenocortical cells. In this regard, Ang II transduction signaling promotes a variety of molecular mechanisms that finely tune steroid hormone production. As shown in **Figure 1**, signal transduction pathways in adrenocortical cells may interact at different signaling levels, and mitochondrial fusion arises as an essential event in full steroid production. Ang II-stimulated steroidogenesis is also mediated by an increase in intracellular AA which is converted into eicosanoids to activate OXER1 and further increase StAR expression, steroid synthesis, and cell migration. Given the undoubted relevance of phospho/dephosphorylation cascades in hormone-dependent signaling, we have reviewed here the regulation of MKPs expression in adrenocortical cells. Particularly, we highlight the hormonal regulation of MKP-1 and MKP-3 in adrenocortical cells and the putative action of these phosphatases in the expression of steroidogenic enzymes. We have also discussed the potential impact of MKP-3 on cell cycle regulation. In conclusion, despite the extensive evidence of adrenocortical cell hormone signaling pathways, molecular mechanisms recently described support the notion that the regulation of adrenal cortex metabolism deserves to be further explored.

## 6 Future perspectives

Regarding mitochondrial fusion, further studies on the mechanisms controlling Mfn2 expression may prove of great interest. Indeed, Mfn2 levels regulate physiological processes such as steroid synthesis, and Mfn2 downregulation has been extensively associated with the onset of human diseases and tumorigenic processes. To date, no information about a correlation between Mfn2 expression and adrenocortical tumor development has been reported. Regulation of OPA1 and Drp1 in adrenal cells requires more exploration, as these proteins are essential for a proper mitochondrial fusion/fission balance. These results may fuel interesting future studies on mitochondrial dynamics in adrenal tissue.

The biological functions of AA-derived lipid mediators remain poorly understood. Because of their characteristics, they can have single-handed effects or else act through receptors, which represents a challenge in discriminating the pathways that mediate their effects on the regulation of cellular functions. Although little is known about the signal transduction pathways initiated by *in vivo* OXER1 activation, studies have been conducted using inhibitory compounds acting differentially on targets after receptor activation or antagonists. The application of high-throughput techniques, the development of specific agonists and antagonists, and the manipulation of gene expression will allow progress in this direction.

Regarding the role of MKPs in steroidogenic tissues, further studies will be necessary to define the possible involvement of MKP-3 in steroid production induced by Ang II. Moreover, our group aims to elucidate not only the participation of MKP-3 in cell proliferation and steroidogenesis but also the relevance of each MKP-3 variant in these processes.

## Author contributions

MMMSG, CP, AFC, PMM, FCM and CeP wrote and revised the manuscript; MMMSG and CeP performed the Figure; MMMSG, CP, AFC, YB, MAB, ABM, PMM, FCM and CeP provided critical revisions of the manuscript. All authors contributed to the article and approved the submitted version.
